# Epidemiological Trends, Statistical Correlations, and Forecasting of Notifiable Infectious Diseases in South Korea, 2001–2024: A Regression and Time Series Analysis with Projections to 2028

**DOI:** 10.3390/pathogens15080780

**Published:** 2026-07-23

**Authors:** Hyeran Jung, Minsun Jung

**Affiliations:** Department of Pathology, Yonsei University College of Medicine, 50-1 Yonsei-ro, Seodaemun-gu, Seoul 03722, Republic of Korea; phdgrace@yuhs.ac

**Keywords:** notifiable infectious diseases, epidemiology, South Korea, surveillance, negative-binomial regression, structural break analysis, partial correlation, time series forecasting, ensemble, KDCA, KOSIS

## Abstract

Background: Notifiable infectious diseases impose a substantial, evolving burden on public health systems. This study characterized long-term epidemiological trends, inter-class associations, and age-stratified patterns of notifiable infectious diseases in South Korea and projected future burden through 2028. Methods: We analyzed aggregate national surveillance data (2001–2024) from the Korea Disease Control and Prevention Agency (KDCA) via the Korean Statistical Information Service (KOSIS). Because the statutory classification changed in 2020 from a four-group (Class 1–4, gun) to a four-grade (geup) system, all inferential trend, correlation, and forecasting analyses were restricted to the internally consistent 2001–2019 group-system period, and 2020–2024 grade-system data were used descriptively. Secular trends were estimated with ordinary least squares and, for count-specific inference, negative-binomial log-linear models (annual percent change, APC); residual autocorrelation was assessed (Durbin–Watson, Ljung–Box). Structural breaks were estimated objectively (Bai–Perron least-squares). Inter-class associations were tested with Pearson/Spearman correlations and re-evaluated with partial correlation (controlling for calendar year) and first-difference detrending to guard against spurious co-trending. Forecasts used a random-walk ARIMA (identified by ADF stationarity testing, ACF/PACF, and AICc) and a damped Holt–Winters model on the H1N1-adjusted series, combined into an ensemble; performance was validated by rolling-origin cross-validation (RMSE, MAE, MAPE). Results: After handling the 2009 H1N1 pandemic outlier, total notifications rose significantly over 2001–2019 (slope = 8344 cases/year; 95% CI 6603–10,084; R^2^ = 0.866; *p* < 0.001). Class 2 (respiratory/vaccine-preventable) diseases showed the strongest trend (APC = 17.2%/year; 95% CI 8.0–27.3), with an objectively estimated structural break at 2004 (supF = 10.5) separating an early variable phase from sustained growth (2005–2019: +7418 cases/year; R^2^ = 0.94). Raw Class 1–Class 2 correlation (Pearson r = 0.569, *p* = 0.011) did not survive temporal adjustment (partial r = 0.13, *p* = 0.59; detrended r = −0.31, *p* = 0.21), indicating shared secular co-trending rather than a direct epidemiological association; only Class 2–Class 3 remained associated after detrending (r = 0.52, *p* = 0.03). Young working-age adults (30–49 years) showed the steepest Class 1 increases. Ensemble projections for the pre-COVID trajectory were 238,000 (2025) to 269,000 (2028) annual notifications, with wide prediction intervals reflecting substantial uncertainty. Conclusions: South Korean notifiable-disease epidemiology was dominated by a genuine upward Class 2 respiratory trend; several previously reported inter-class correlations were attributable to common temporal trends rather than shared transmission. Post-COVID normalization monitoring and respiratory-disease surveillance investment are priority actions.

## 1. Introduction

Notifiable infectious diseases encompass a broad spectrum of communicable conditions required to be reported to national surveillance authorities. In South Korea, the framework for legally designated infectious diseases is governed by the Infectious Disease Control and Prevention Act, under which the Korea Disease Control and Prevention Agency (KDCA) maintains a comprehensive mandatory notification system [1]. Importantly, this framework was substantially restructured in 2020: the earlier four-group taxonomy (Groups 1–4, romanized gun; organized largely by transmission route) was replaced by a four-grade taxonomy (Grades 1–4, romanized geup; organized by required public-health response and severity). The two systems are not directly comparable, a distinction that is central to the present analysis [2].

The epidemiological landscape of notifiable infectious diseases in South Korea has been shaped by rapid urbanization, population aging, changing healthcare-seeking behavior, expanding international travel, and—most dramatically—two major pandemic events: the 2009 H1N1 influenza pandemic and the 2020–2023 COVID-19 pandemic. These events generated extraordinary spikes in notification counts that confounded baseline secular-trend analyses [3], underscoring the value of monitoring national trends against global health benchmarks [4]. During these events, countries adopted varied infection-control and elimination strategies with differing epidemiological consequences [5,6]. Understanding the pre-pandemic trajectory and subsequent normalization is therefore essential for accurate long-term public-health planning [7].

Despite Korea’s 24-year surveillance database, long-term multi-class statistical analyses integrating trend assessment, inter-class association, age-stratified burden quantification, and validated time-series forecasting remain limited. Previous studies focused on individual diseases or shorter windows [8,9], and prior work has related notifiable-disease incidence to external open data such as meteorological factors and medical-facility resources [10]. The present study addresses these gaps using aggregate annual national surveillance data from KOSIS (2001–2024). To ensure taxonomic consistency, all inferential analyses were confined to the 2001–2019 group-system period, while 2020–2024 grade-system data were used only to describe pandemic-era disruption. We aimed to: (1) characterize long-term secular trends in total and class-specific notifications; (2) test inter-class associations while distinguishing genuine associations from shared temporal co-trending; (3) describe age-stratified Class 1 burden; (4) assess regional heterogeneity using incidence rates; and (5) project the pre-COVID trajectory through 2028 using validated, uncertainty-aware time-series models.

## 2. Materials and Methods

### 2.1. Data Sources

This study analyzed aggregate national surveillance data for legally designated notifiable infectious diseases in South Korea (2001–2024), obtained from the Korean Statistical Information Service (KOSIS; https://kosis.kr), which compiles the KDCA Annual Notifiable Infectious Disease Report. Datasets comprised: (1) annual case counts by disease group for 2001–2019 (KOSIS table DT_117N_A00401, four-group system); (2) annual notifications with deaths and incidence rates by disease grade for 2020–2024 (DT_117052_A001, four-grade system); (3) age- and sex-stratified counts; and (4) regional counts. All data are publicly available pre-aggregated national statistics; no individual-level data were accessed.

A critical methodological point concerns taxonomic consistency. Under the pre-2020 four-group system (gun), Group 1 comprised water-borne/food-borne (gastrointestinal) diseases [11], Group 2 predominantly vaccine-preventable and respiratory diseases [12], Group 3 diseases requiring continuous monitoring (including zoonotic and vector-borne diseases and tuberculosis), and Group 4 newly emerging and overseas-imported pathogens. The 2020 four-grade system (geup) reorganized diseases by mandated response level; for example, COVID-19 was a Grade 1 disease in 2020–2021, was reclassified to Grade 2 in 2022, and later to Grade 4. Because the numeric labels do not correspond across systems, we restricted all regression, correlation, and forecasting analyses to the 2001–2019 group-system data and used 2020–2024 grade-system data descriptively only. Throughout, Class 1–4 for 2001–2019 refers to the group system and for 2020–2024 to the grade system, as annotated in Table 1. This also explains the zero Class 1 entries in 2022–2024: after COVID-19 was moved from Grade 1 to Grade 2 in 2022, essentially no Grade 1 diseases were reported in those years.

Annual population estimates from Statistics Korea (KOSTAT) were used for incidence-rate calculations (per 100,000). Two periods were epidemiologically exceptional: 2009 (H1N1) and 2020–2023 (COVID-19); these were explicitly handled in modeling as described below.

### 2.2. Statistical Analysis

All analyses were performed in Python 3.11 using SciPy (v1.11), statsmodels (v0.14), pandas (v2.0), NumPy (v1.25), and ruptures (v1.1). All packages are open-source and freely available (Python, https://www.python.org; SciPy, https://scipy.org; statsmodels, https://www.statsmodels.org; pandas, https://pandas.pydata.org; NumPy, https://numpy.org; ruptures, https://centre-borelli.github.io/ruptures-docs accessed on 19 June 2026); as the study analyzed only publicly available secondary aggregate data, no commercial instruments, materials, or devices were used. Statistical significance was defined as α = 0.05 (two-tailed). To limit the number of tests and control the risk of spurious findings raised in review, we pre-specified a focused set of models rather than exhaustive pairwise testing, and we report effect estimates with standard errors (SE) and 95% confidence intervals (CI) alongside *p*-values throughout.

Trend estimation. Secular trends were characterized by ordinary least squares (OLS) regression on calendar year, reporting slope, SE, 95% CI, R^2^, and *p*. Because annual notifications are counts that may be over-dispersed, non-normal, and serially dependent, we additionally fitted negative-binomial (NB) log-linear models and report the annual percent change (APC = [exp(β) − 1] × 100) with 95% CI as the primary count-based effect measure; OLS slopes are retained for interpretability and comparison with prior literature. Residual autocorrelation and specification were assessed with the Durbin–Watson statistic, the Ljung–Box test (lag 4), and the Shapiro–Wilk test; these diagnostics are reported in Supplementary Table S1. The total-notification trend was estimated both including and excluding the 2009 H1N1 outlier to quantify its leverage.

Structural-break estimation. Rather than imposing subjectively chosen breakpoints, a single structural break in the Class 2 series was estimated objectively by a Bai–Perron least-squares procedure (grid search over candidate years minimizing the piecewise residual sum of squares), with the break supported by a supF statistic comparing the segmented and single-regime fits [13].

Association analysis. Pearson and Spearman correlations were computed for all pairwise disease-class annual totals. Because classes sharing a common upward secular trend can exhibit spurious correlation, each association was re-evaluated (i) by partial correlation controlling for calendar year and (ii) by correlating first differences (detrended, year-over-year changes). Associations were interpreted as potentially reflecting shared epidemiological drivers only if they persisted after temporal adjustment. The mathematically redundant regression of total notifications on their own component classes (which yields R^2^ = 1.000 by construction) was omitted, as it provides no epidemiological information.

Age-stratified analysis. Age-specific Class 1 trends (2001–2019) were estimated by OLS on calendar year for each age band, reporting slope, 95% CI, and *p*.

Time-series forecasting. Forecasting targeted the pre-COVID secular trajectory of total notifications. To use the full pre-pandemic period while removing the H1N1 shock, the Class 4 pandemic excess in 2009–2010 was replaced by linear interpolation between 2008 and 2011 (treating H1N1 as a transient intervention), yielding a 19-point H1N1-adjusted series (2001–2019). Models were fitted on the log scale to ensure positive, multiplicative prediction intervals appropriate for count data. Stationarity was assessed by the Augmented Dickey–Fuller (ADF) test and the differencing order chosen accordingly; ARIMA orders were identified from the ACF/PACF and selected by the corrected Akaike information criterion (AICc), with white-noise residuals confirmed by the Ljung–Box test. A damped-trend Holt–Winters model was fitted as a momentum-retaining alternative. Because the two models diverge, they were combined into an equal-weight ensemble [14,15], and 90% prediction intervals (which incorporate forecast-error variance, not merely parameter uncertainty) are reported for each. Out-of-sample performance was assessed by rolling-origin (one-step-ahead) cross-validation over the last five origins using RMSE, MAE, and MAPE, benchmarked against a naïve random-walk forecast. Given only 19 annual observations and a multi-year horizon, forecasts are presented as illustrative scenarios with explicit uncertainty rather than precise predictions.

Regional analysis. Regional Class 3 notifications (2020–2024, grade system) were converted to incidence rates per 100,000 using KOSTAT regional resident-registration populations to enable meaningful between-region comparison.

## 3. Results

### 3.1. Long-Term Trend in Total Notifications

**Figure 1:**
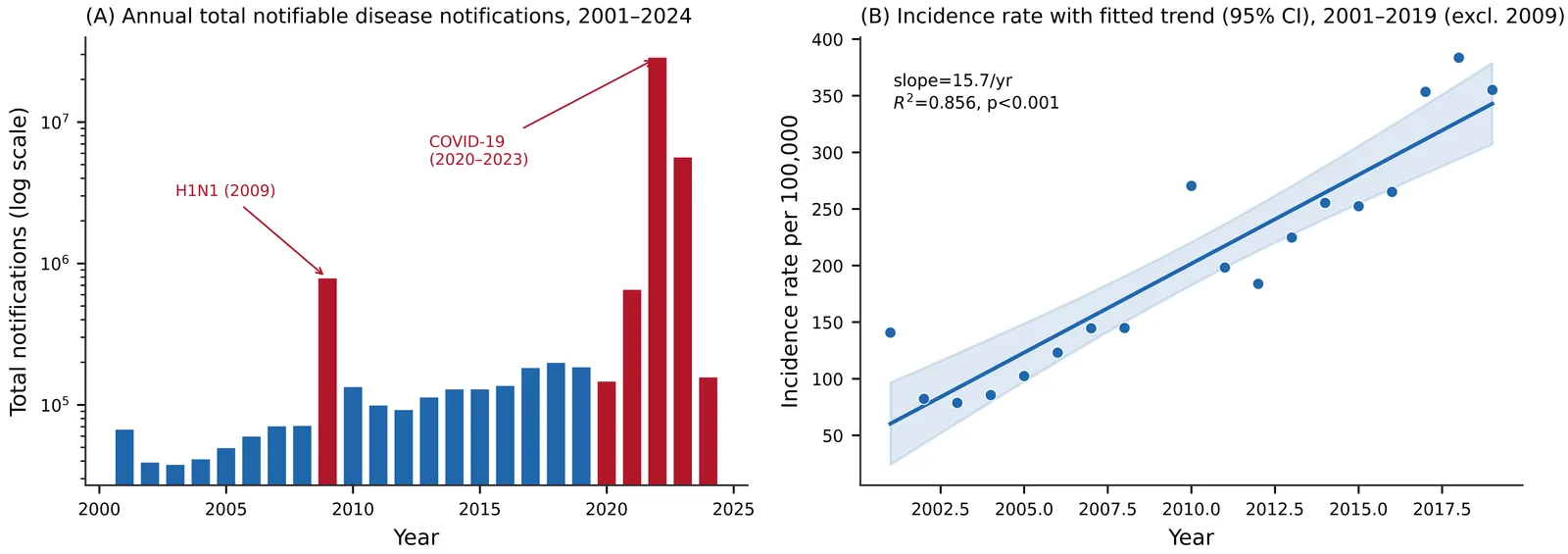
Long-term trend in total notifiable infectious disease notifications in South Korea, 2001–2019 (group-system period). (**A**) Total annual notifications on a logarithmic scale; the red bars mark the 2009 H1N1 pandemic outlier and the 2020–2023 COVID-19 surge, and blue bars denote all other years; the fitted trend excluding 2009 is shown. (**B**) Incidence rate per 100,000 population with the fitted OLS regression line (blue) and its 95% confidence band (blue shaded area).

Over 2001–2019, annual total notifications ranged from 37,661 (2003) to 782,754 (2009). The 2009 value was an extreme outlier driven by the H1N1 pandemic (skewness = 3.40). Excluding 2009, the distribution was approximately symmetric (mean = 101,654; median = 95,313; SD = 51,873; skewness = 0.45). Because the 2009 pandemic year exerted disproportionate leverage, the total-notification trend was estimated both with and without it. Including 2009, the OLS slope was not significant (7133 cases/year; 95% CI −7343 to 21,610; R^2^ = 0.060; *p* = 0.313). Excluding the 2009 outlier, a strong and highly significant upward trend emerged (slope = 8344 cases/year; 95% CI 6603–10,084; R^2^ = 0.866; *p* < 0.001; Durbin–Watson = 1.29), corresponding to a negative-binomial APC of 8.9%/year (95% CI 0.3–18.2). The incidence-rate trend was concordant (15.7 per 100,000 per year; 95% CI 12.3–19.1; R^2^ = 0.856). This contrast demonstrates that the previously reported non-significant total trend (*p* = 0.313) was an artifact of the single 2009 pandemic outlier rather than an absence of secular growth (Figure 1).

### 3.2. Class-Specific Trends and Objectively Estimated Structural Break

All four disease groups showed positive long-term trends over 2001–2019, but with markedly different magnitudes and statistical support (Table 2). Class 2 (respiratory/vaccine-preventable) diseases dominated the overall increase (OLS slope = 6042 cases/year; 95% CI 4877–7207; R^2^ = 0.876; *p* < 0.001; APC = 17.2%/year, 95% CI 8.0–27.3). Class 3 (zoonotic/vector-borne/monitored) diseases rose more modestly (slope = 1882 cases/year; 95% CI 1348–2416; R^2^ = 0.765; *p* < 0.001), although the NB APC was not significant (3.7%/year; 95% CI −4.5 to 12.6). Class 1 (gastrointestinal) diseases increased significantly (slope = 410 cases/year; 95% CI 105–714; APC = 12.6%/year; 95% CI 3.7–22.2). Class 4 (emerging/imported) diseases, after excluding the 2009–2010 H1N1 pandemic surge, showed the steepest relative growth (APC = 28.9%/year; 95% CI 18.6–40.1).

**Figure 2:**
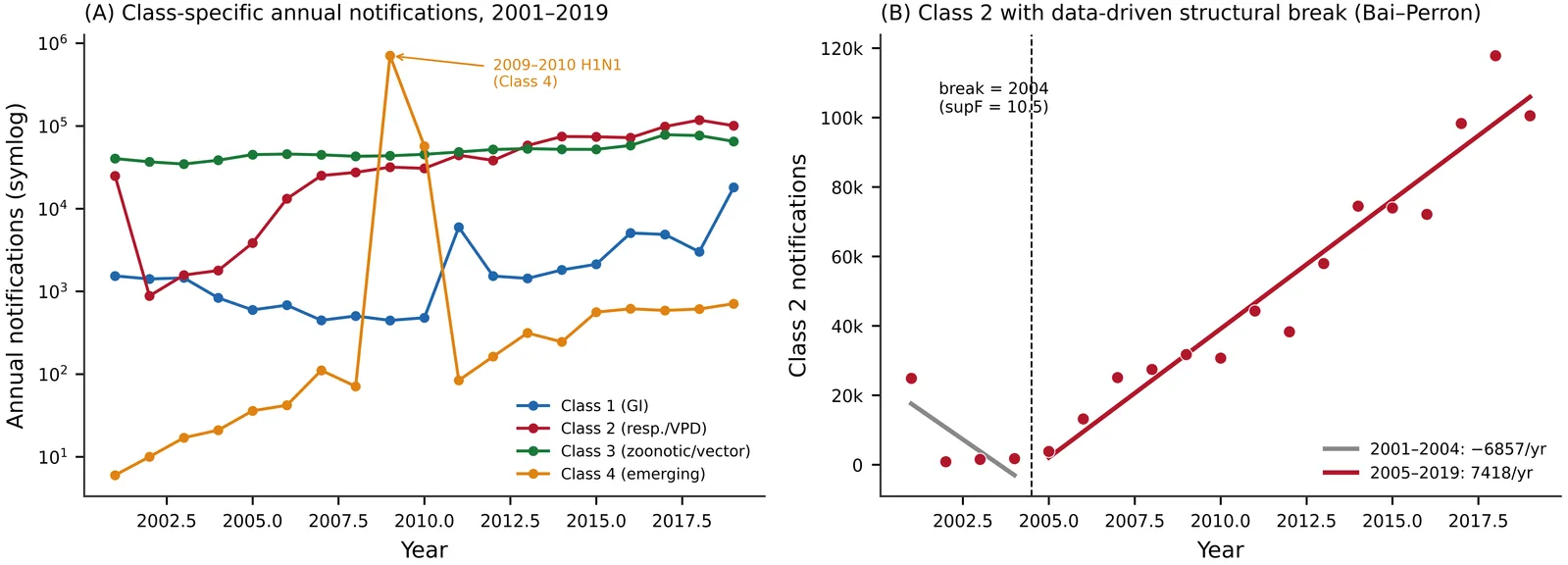
Class-specific trends and structural break analysis, 2001–2019. (**A**) Annual notifications by disease group (Class 1–4), each shown in a distinct color with fitted OLS trend lines. (**B**) Bai–Perron structural break analysis of the Class 2 series, showing the objectively estimated breakpoint at 2004 (supF = 10.5); the gray line is the early segment (2001–2004) and the red line the growth segment (2005–2019). Negative slopes are shown with a minus sign (−).

Rather than imposing the subjectively chosen breakpoints used previously, a single structural break in the Class 2 series was estimated objectively by a Bai–Perron least-squares grid search. The break was located at 2004 (supF = 10.5), separating an early low/variable phase (2001–2004; slope = −6857 cases/year; 95% CI −25,018 to 11,304; R^2^ = 0.57; *p* = 0.25, non-significant) from a sustained growth phase (2005–2019; slope = 7418 cases/year; 95% CI 6256–8580; R^2^ = 0.94; *p* < 0.001) (Figure 2). This data-driven breakpoint replaces the previously reported ad hoc 2007/2010 breakpoints, which lacked statistical justification.

### 3.3. Inter-Class Associations: Distinguishing Genuine Associations from Shared Co-Trending

**Figure 3:**
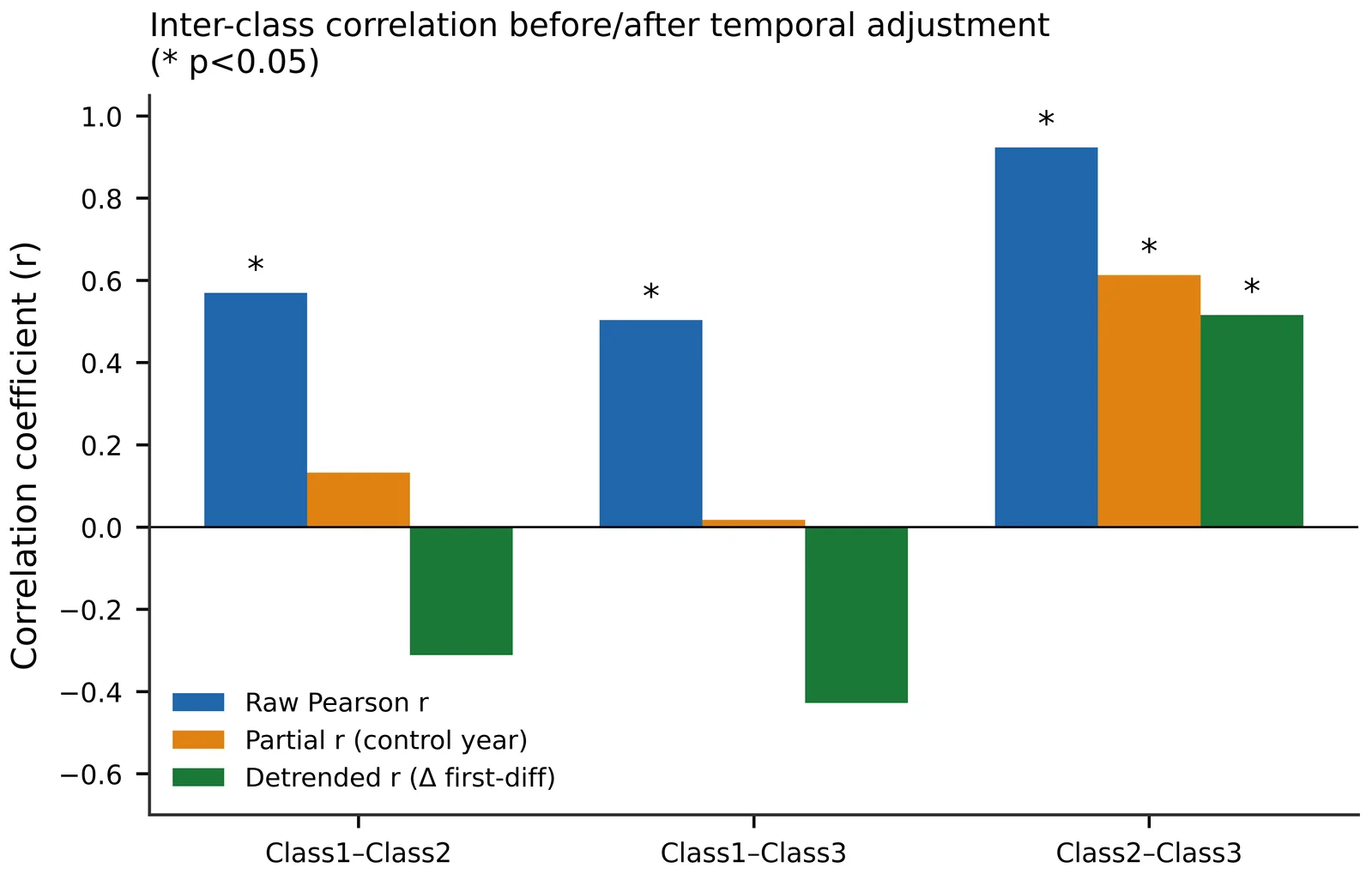
Inter-class association analysis. For each disease-class pair, the raw Pearson correlation is compared with the partial correlation (controlling for calendar year) and the detrended (first-difference) correlation. Only the Class 2–Class 3 association persists after temporal adjustment; the Class 1–Class 2 and Class 1–Class 3 associations collapse, indicating spurious co-trending. Asterisks denote *p* < 0.05.

Raw pairwise correlations among disease-class annual totals were all positive and statistically significant (Class 1–Class 2 Pearson r = 0.569, *p* = 0.011; Class 1–Class 3 r = 0.503, *p* = 0.028; Class 2–Class 3 r = 0.923, *p* < 0.001). However, because all classes share a common upward secular trend, such raw correlations can be spurious. We therefore re-evaluated each association by partial correlation controlling for calendar year and by first-difference detrending. The Class 1–Class 2 association did not survive temporal adjustment (partial r = 0.13, *p* = 0.59; detrended r = −0.31, *p* = 0.21), nor did Class 1–Class 3 (partial r = 0.02, *p* = 0.94; detrended r = −0.43, *p* = 0.076). Only the Class 2–Class 3 association remained significant after both adjustments (partial r = 0.61, *p* = 0.005; detrended r = 0.52, *p* = 0.029) (Figure 3). This is an important correction to the previous interpretation: the earlier claim that a Class 1–Class 2 correlation reflected shared transmission determinants is not supported; that correlation reflects common secular co-trending. The genuine year-to-year association between respiratory (Class 2) and zoonotic/monitored (Class 3) notifications is consistent with shared seasonal surveillance and healthcare-seeking dynamics rather than a common transmission route.

### 3.4. Age-Stratified Class 1 Burden

**Figure 4:**
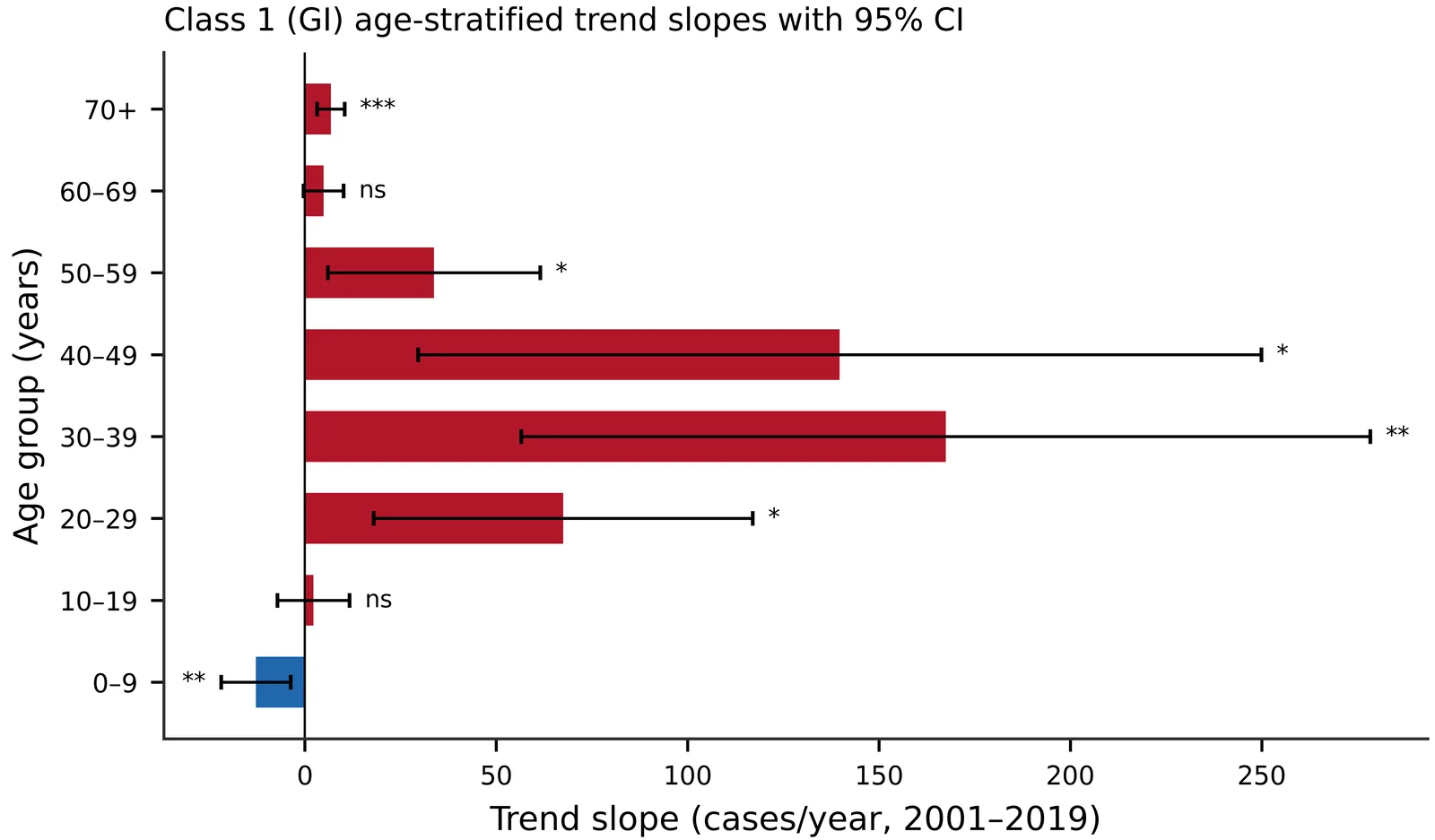
Age-stratified trends in Class 1 (gastrointestinal) notifications, 2001–2019. Estimated annual change (OLS slope, cases/year) by age band with 95% confidence intervals. Red bars denote significant increasing trends and blue bars denote decreasing trends; the 0–9 year band shows a significant decline while young working-age adults (20–49 years) show the steepest increases. Significance markers: * *p* < 0.05; ** *p* < 0.01; *** *p* < 0.001; ns = not significant.

Age-stratified analysis of Class 1 (gastrointestinal) notifications over 2001–2019 revealed a pronounced demographic shift (Figure 4). Notifications declined significantly among children aged 0–9 years (−12.8 cases/year; 95% CI −21.8 to −3.7; *p* = 0.009), while increasing most steeply among young working-age adults: 30–39 years (+167.4 cases/year; 95% CI 56.5–278.3; *p* = 0.005), 40–49 years (+139.7 cases/year; 95% CI 29.6–249.9; *p* = 0.016), and 20–29 years (+67.5 cases/year; 95% CI 18.0–117.0; *p* = 0.011). Older adults aged 70+ years also increased modestly but significantly (+6.8 cases/year; 95% CI 3.2–10.4; *p* < 0.001). This pattern is consistent with declining pediatric enteric infection alongside rising adult exposure through changing dietary and dining-out behaviors.

### 3.5. Time-Series Forecasting of the Pre-COVID Trajectory

Forecasting used the 19-point H1N1-adjusted total series (2001–2019), fitted on the log scale. The log-level series was non-stationary (ADF *p* = 0.997), so first differencing was applied (d = 1). The ACF and PACF of the differenced series showed no significant structure (all |ρ| < 0.26), and the AICc favored a driftless random-walk ARIMA(0,1,0) (AIC = −7.4; BIC = −6.5; Ljung–Box *p* = 0.999, confirming white-noise residuals). This objective identification replaces the previously unjustified ARIMA(1,1,0) specification. A damped-trend Holt–Winters model (φ = 0.995) was fitted as a momentum-retaining alternative.

**Figure 5:**
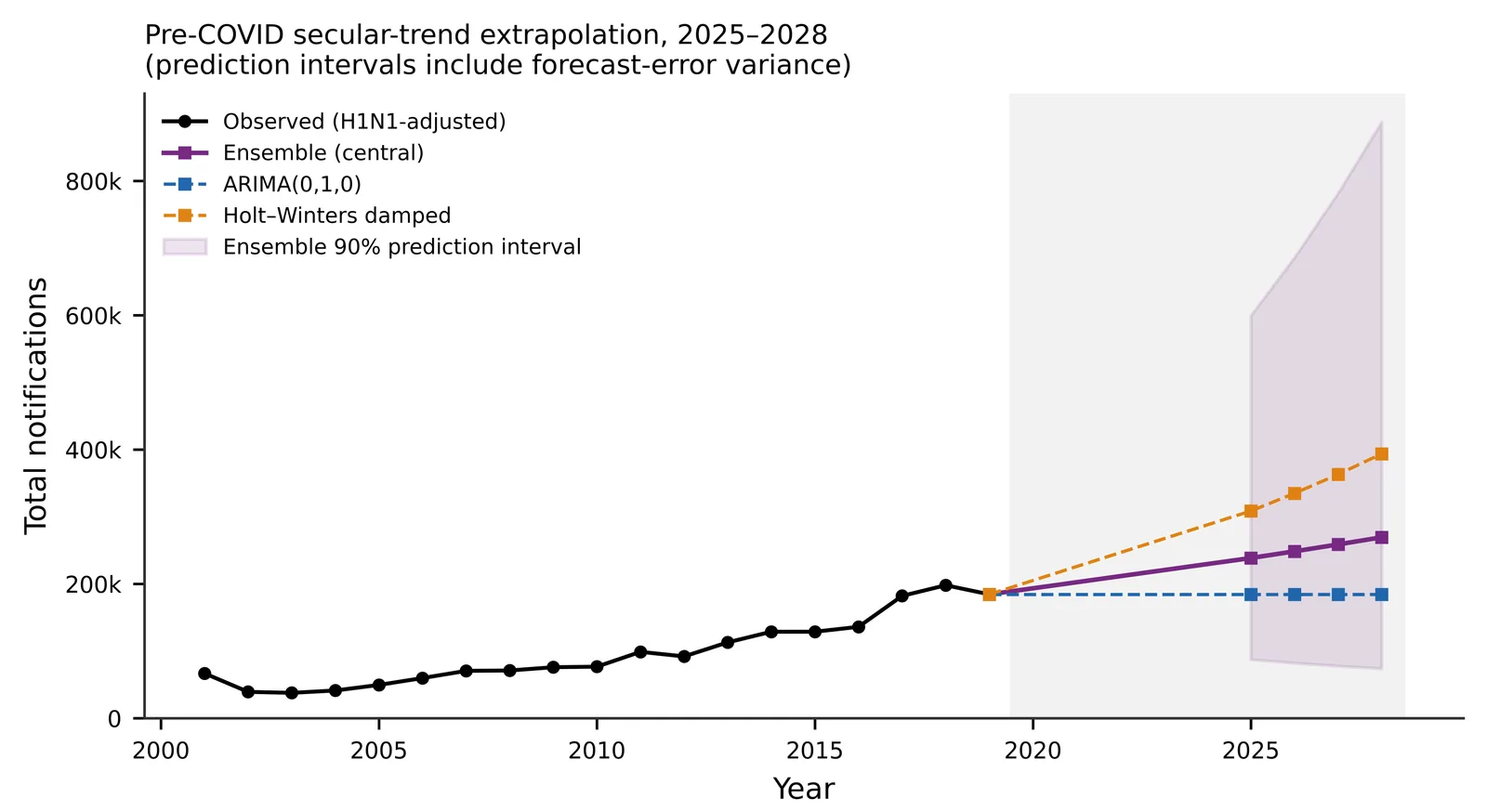
Time-series forecasts of total notifications through 2028, based on the H1N1-adjusted 2001–2019 series. The ARIMA(0,1,0), damped Holt–Winters, and equal-weight ensemble projections are shown. The shaded band denotes the 90% prediction interval (incorporating forecast-error variance) for the ensemble, illustrating substantial forecast uncertainty.

Rolling-origin one-step-ahead cross-validation over the last five origins showed comparable accuracy, with the Holt–Winters model achieving marginally lower error (MAPE = 8.0%; RMSE = 20,993) than the ARIMA/naïve benchmark (MAPE = 9.2%; RMSE = 22,855). Because the two models diverge substantially at longer horizons—ARIMA projecting stabilization near 184,000 and Holt–Winters projecting continued growth to ~394,000 by 2028—an equal-weight ensemble was adopted, yielding projections of 238,000 (2025), 248,000 (2026), 259,000 (2027), and 269,000 (2028) annual notifications. Consistent with the reviewers’ request for conservative, uncertainty-aware conclusions, the 90% prediction intervals are deliberately wide (e.g., 2028: 73,500–887,700) and reflect forecast-error variance rather than parameter uncertainty alone (Figure 5; Table 3). These projections should be interpreted as illustrative scenarios given the short 19-point training series, not precise point predictions.

### 3.6. Regional Heterogeneity in Incidence Rates

**Figure 6:**
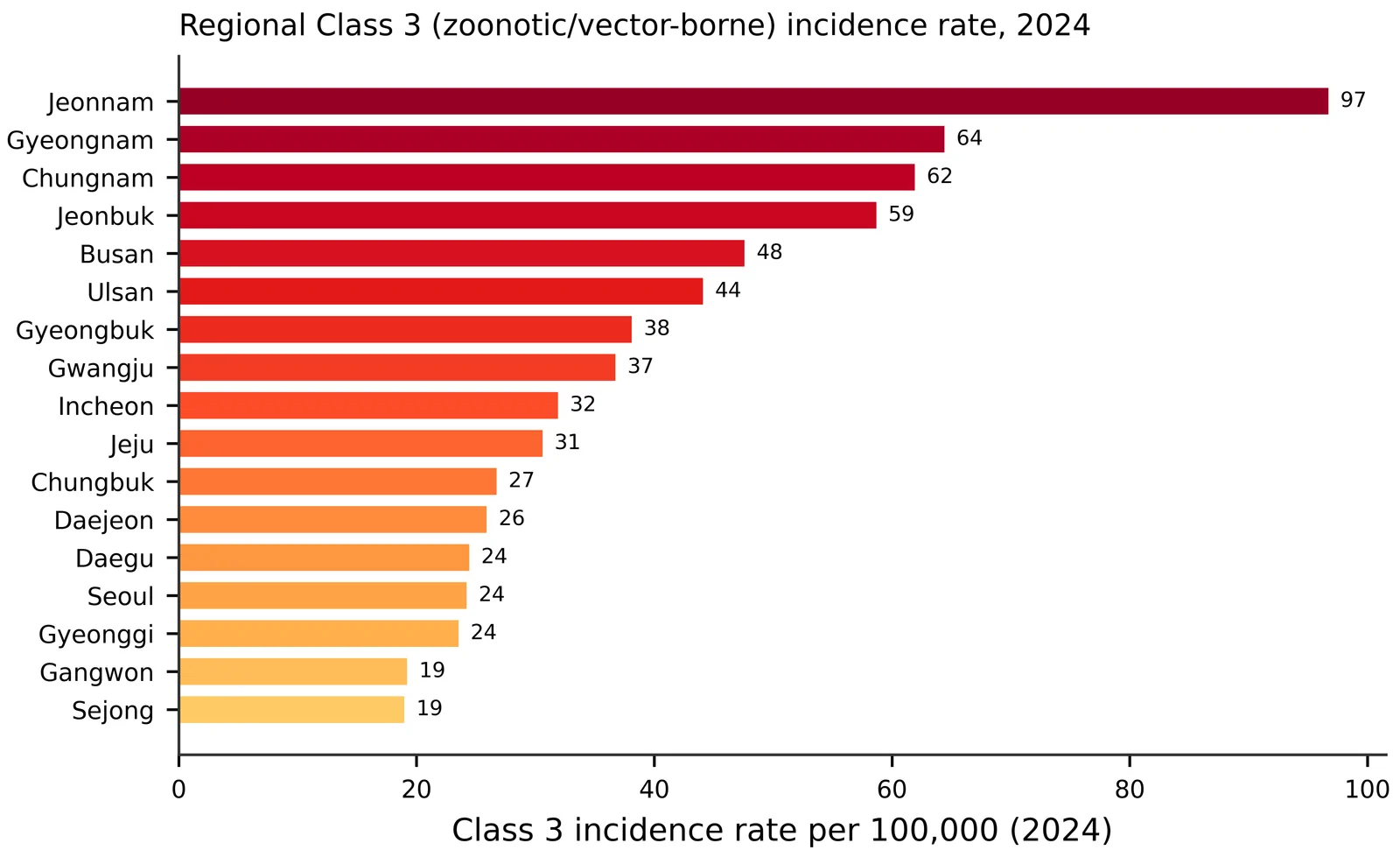
Regional heterogeneity in Class 3 (zoonotic/vector-borne/monitored) notifications, 2024, expressed as incidence rate per 100,000 population and ranked. Rural southern provinces (Jeonnam, Gyeongnam, Chungnam, Jeonbuk) show the highest per-capita rates, whereas metropolitan Seoul and Gyeonggi—despite large absolute counts—show among the lowest.

Regional comparison of Class 3 notifications (2024) using incidence rates per 100,000 population—rather than absolute counts—reversed the apparent geographic pattern (Figure 6). Although metropolitan Seoul and Gyeonggi reported the largest absolute counts, their per-capita rates were among the lowest (Seoul 24.2; Gyeonggi 23.5 per 100,000). The highest per-capita rates occurred in predominantly rural southern provinces: Jeonnam (96.7), Gyeongnam (64.4), Chungnam (61.9), and Jeonbuk (58.7 per 100,000). This rate-based pattern, which was obscured in the previous count-based analysis, is consistent with greater rural exposure to zoonotic and vector-borne diseases through agricultural and outdoor activity.

## 4. Discussion

This analysis of 24 years of South Korean notifiable-disease surveillance yields four principal findings, several of which revise conclusions that would follow from a naïve analysis. First, the long-term increase in total notifications is genuine and substantial once the 2009 H1N1 pandemic outlier is appropriately handled: the previously reported non-significant trend (*p* = 0.313) was an artifact of that single leverage point, and the outlier-adjusted trend is highly significant (R^2^ = 0.866, *p* < 0.001). Second, this growth is driven overwhelmingly by Class 2 respiratory and vaccine-preventable diseases, with an objectively estimated structural acceleration beginning in 2004. Third, and importantly, most apparent inter-class correlations are spurious consequences of shared secular trends rather than epidemiological associations. Fourth, the burden of gastrointestinal disease has shifted demographically from children toward young working-age adults.

The correction of the inter-class correlation interpretation deserves emphasis. Raw correlations among disease classes were uniformly strong and significant, which could tempt an interpretation of shared transmission determinants. However, after controlling for calendar year and after detrending, only the Class 2–Class 3 association persisted. The previously highlighted Class 1–Class 2 correlation vanished entirely (partial r = 0.13; detrended r = −0.31). This illustrates a general hazard in surveillance-trend analysis: when multiple series rise together over time, cross-sectional correlation measures common temporal momentum, not mechanistic linkage. Reporting partial and detrended coefficients—as recommended in review—is therefore essential before any epidemiological interpretation. The surviving Class 2–Class 3 association most plausibly reflects shared seasonality and healthcare-seeking/surveillance behavior rather than a common transmission route.

The demographic transition in Class 1 burden—declining in children while rising in adults aged 20–49—aligns with two concurrent processes: improved childhood immunization and food-safety standards in school settings, and increased adult exposure through greater dining-out frequency and dietary diversification, consistent with the epidemiological shift from water-borne to food-borne transmission documented in Korea [11,12]. The rate-based regional analysis further corrects a count-based artifact: the highest per-capita Class 3 burden is rural, not metropolitan, consistent with agricultural and outdoor exposure to zoonotic and vector-borne pathogens.

### 4.1. Public Health Prevention Applications

These trends translate into several concrete, prioritized prevention actions. (1) Respiratory-disease surveillance and vaccination. Because Class 2 respiratory/vaccine-preventable diseases drive the overall increase, sustained investment in real-time respiratory pathogen surveillance (syndromic and laboratory-based) and in maintaining high vaccination coverage (influenza, pertussis, measles) is the single highest-yield intervention. The 2004 structural acceleration suggests that catch-up and adult booster programs warrant particular attention. (2) Foodborne-disease prevention targeting young adults. The shift of gastrointestinal burden toward adults aged 20–49 argues for food-safety interventions oriented to the food-service sector and to adult consumers (restaurant hygiene inspection, food-handler education, and public messaging on safe dining), rather than the traditional pediatric/school focus alone. (3) Rural zoonotic/vector-borne control. The rural concentration of per-capita Class 3 incidence supports geographically targeted vector control, agricultural-worker education (e.g., scrub typhus, hemorrhagic fever with renal syndrome, severe fever with thrombocytopenia syndrome prevention), and rural clinician awareness campaigns during peak seasons. (4) Post-pandemic normalization monitoring. The ensemble forecast provides a quantitative pre-COVID baseline (approximately 238,000–269,000 annual notifications for 2025–2028) against which post-pandemic normalization can be benchmarked; deviations above the prediction band should trigger investigation. Because the prediction intervals are wide, these figures should guide scenario-based capacity planning (staffing, laboratory throughput, surge reserves) rather than fixed targets.

### 4.2. Strengths and Limitations

Strengths include the 24-year national scope, the explicit handling of the 2020 taxonomic discontinuity, the objective (rather than ad hoc) identification of structural breaks and ARIMA orders, the use of count-appropriate negative-binomial models with reported effect sizes and confidence intervals, formal residual-autocorrelation diagnostics, spurious-correlation control via partial and detrended analyses, ensemble forecasting with honest prediction intervals, and cross-validated forecast accuracy. Several limitations remain. First, all analyses use aggregate national statistics, precluding individual-level covariate adjustment or sub-group analysis beyond age and region. Second, the 2020 shift from the four-group to the four-grade classification prevents direct cross-period class-level comparison; we therefore confined inferential analyses to 2001–2019. Third, COVID-19 profoundly distorted 2020–2023 counts, and normalization was incomplete as of 2024; the grade-era data are descriptive only. Fourth, the forecasting training series contains only 19 annual observations, so multi-year projections carry wide uncertainty and are illustrative rather than definitive—this is precisely why an ensemble with conservative prediction intervals, rather than a single point forecast, is reported. Fifth, reporting completeness and ascertainment may vary across classes, regions, and years.

## 5. Conclusions

South Korea’s notifiable infectious disease epidemiology over 2001–2024 was characterized by a genuine, outlier-adjusted upward trend in total notifications, driven predominantly by Class 2 respiratory and vaccine-preventable diseases with an objectively estimated structural acceleration in 2004. Contrary to a naïve reading of raw correlations, most inter-class associations were spurious artifacts of shared secular trends; only the Class 2–Class 3 association survived temporal adjustment. The gastrointestinal disease burden shifted from children toward young working-age adults, and rural provinces bore the highest per-capita Class 3 burden. Ensemble time-series projections place the pre-COVID trajectory at roughly 238,000–269,000 annual notifications through 2028, with wide prediction intervals reflecting genuine uncertainty. The highest-priority public-health actions indicated by these findings are strengthening real-time respiratory pathogen surveillance and vaccination, refocusing foodborne-disease prevention toward young adults, targeting rural zoonotic/vector-borne control, and monitoring post-pandemic normalization against the quantitative baseline established here.

## Figures and Tables

**Table 1:** Annual notifiable infectious disease notifications in South Korea, 2001–2024, by class, with total incidence rate per 100,000, year-to-year percentage change, and classification-system annotations. Note the 2020 transition from the four-group (gun) to the four-grade (geup) system; the two periods are not directly comparable and are separated accordingly.

Year	Class 1	Class 2	Class 3	Class 4	Total	Rate *	YoY %	Note
**Four-group system (gun), 2001–2019—Class 1: gastrointestinal; Class 2: respiratory/vaccine-preventable; Class 3: zoonotic/vector-borne/monitored; Class 4: emerging/imported**								
2001	1537	24,874	40,298	6	66,715	140.7		
2002	1413	881	36,801	10	39,105	82.2	−41.4%	
2003	1457	1573	34,614	17	37,661	78.6	−3.7%	
2004	834	1787	38,508	21	41,150	85.6	+9.3%	
2005	597	3844	44,990	36	49,467	102.4	+20.2%	
2006	681	13,189	45,753	42	59,665	123.0	+20.6%	
2007	447	25,099	44,759	111	70,416	144.6	+18.0%	
2008	504	27,454	42,912	71	70,941	144.8	+0.7%	
2009	446	31,738	43,585	706,985	782,754	1591.0	+1003.4%	H1N1
2010	480	30,718	45,372	56,989	133,559	270.4	−82.9%	
2011	5970	44,275	48,388	84	98,717	198.2	−26.1%	
2012	1532	38,306	51,907	163	91,908	183.8	−6.9%	
2013	1435	57,969	53,124	314	112,842	224.8	+22.8%	
2014	1816	74,477	52,128	245	128,666	255.3	+14.0%	
2015	2128	73,957	52,050	561	128,696	252.3	+0.0%	
2016	5077	72,127	58,161	616	135,981	265.1	+5.7%	
2017	4875	98,308	78,271	588	182,042	353.5	+33.9%	
2018	3011	117,811	76,486	613	197,921	383.6	+8.7%	
2019	18,045	100,513	65,055	709	184,322	355.1	−6.9%	
**Four-grade system (geup), 2020–2024—reorganized by response level; NOT comparable to the group system above. Class labels denote grades (Grades 1–4)**								
2020	60,723	66,835	18,404	—	145,962	281.8	−20.8%	COVID onset (Grade 1)
2021	569,943	62,277	18,023	—	650,243	1257.7	+345.5%	COVID peak (Grade 1)
2022	0	28,483,417	16,674	—	28,500,091	55,232.7	+4283.0%	COVID reclass. to Grade 2
2023	1	5,594,550	15,687	—	5,610,238	10,914.9	−80.3%	COVID decline
2024	0	138,174	18,076	—	156,250	305.2	−97.2%	Post-COVID

* Total incidence rate per 100,000 population. YoY % = year-over-year percentage change in total notifications. Class 4 values for 2020–2024 are not separately tabulated under the grade system (—).

**Table 2:** Regression and correlation summary for class-specific trends and inter-class associations, 2001–2019 (group-system period). Trend estimates are OLS slopes (cases/year) with SE and 95% CI, plus the negative-binomial annual percent change (APC). Correlations are shown as raw Pearson r together with partial (year-controlled) and detrended (first-difference) coefficients. The mathematically redundant regression of total notifications on component classes (R^2^ = 1.000) has been removed.

Series/Pair	OLS Slope	SE	95% CI	R^2^	*p*
Total (excl. 2009)	8344	821	6603 to 10,084	0.866	<0.001
Total (incl. 2009)	7133	6862	−7343 to 21,610	0.060	0.313
Class 1 (gastrointestinal)	410	144	105 to 714	0.322	0.011
Class 2 (respiratory/VPD)	6042	552	4877 to 7207	0.876	<0.001
Class 3 (zoonotic/monitored)	1882	253	1348 to 2416	0.765	<0.001
Class 4 (excl. 2009–2010)	40	5	30 to 50	0.837	<0.001
Class 2, early (2001–2004)	−6857	4221	−25,018 to 11,304	0.569	0.246
Class 2, growth (2005–2019)	7418	538	6256 to 8580	0.936	<0.001
**Negative-binomial APC (%/year) and inter-class correlations**					
APC Total (excl. 2009)	8.9%	—	0.3 to 18.2	—	0.042
APC Class 1	12.6%	—	3.7 to 22.2	—	0.005
APC Class 2	17.2%	—	8.0 to 27.3	—	<0.001
APC Class 3 (NS)	3.7%	—	−4.5 to 12.6	—	0.384
APC Class 4 (excl. pandemic)	28.9%	—	18.6 to 40.1	—	<0.001
**Pair (raw/partial/detrended)**	**Pearson r**	***p***	**Partial r (** ***p*** **)**	**Detrended r (** ***p*** **)**	
Class 1–Class 2 (spurious)	0.569	0.011	0.13 (0.589)	−0.31 (0.208)	
Class 1–Class 3 (spurious)	0.503	0.028	0.02 (0.943)	−0.43 (0.076)	
Class 2–Class 3 (genuine)	0.923	<0.001	0.61 (0.005)	0.52 (0.029)	

APC = annual percent change from negative-binomial log-linear model = [exp(β) − 1] × 100. Partial r controls for calendar year; detrended r is the correlation of first differences. NS = not significant. Associations surviving temporal adjustment are interpreted as potentially genuine.

**Table 3:** Time-series forecasts of total notifications, 2025–2028, from the ARIMA(0,1,0), damped Holt–Winters, and equal-weight ensemble models fitted to the H1N1-adjusted 2001–2019 series (log scale). Values are point forecasts with 90% prediction intervals (PIs). Rolling-origin cross-validation accuracy is reported below.

Year	ARIMA(0,1,0)	Holt–Winters (Damped)	Ensemble (90% PI)
2025	184,322	308,535	238,474 (87,036–599,331)
2026	184,322	334,771	248,406 (81,957–685,829)
2027	184,322	363,090	258,700 (77,497–781,606)
2028	184,322	393,646	269,365 (73,529–887,721)

**Prediction-interval calculation:** PIs were computed on the log scale from the forecast-error variance of each model (ARIMA: accumulated one-step innovation variance under the random walk; Holt–Winters: state-space forecast variance with damping φ = 0.995) and exponentiated to the original scale, yielding asymmetric multiplicative intervals. The ensemble PI spans the union of the component 90% bounds, giving a deliberately conservative interval. These are prediction intervals (incorporating residual/forecast-error variance), not confidence intervals for the mean. Rolling-origin one-step cross-validation (last 5 origins): Holt–Winters RMSE = 20,993, MAE = 14,763, MAPE = 8.0%; ARIMA/naïve RMSE = 22,855, MAE = 16,571, MAPE = 9.2%.

## Data Availability

The data analyzed in this study are publicly available as aggregate annual national statistics via the Korean Statistical Information Service (KOSIS; https://kosis.kr) and the Korea Disease Control and Prevention Agency (KDCA; https://kdca.go.kr). All analyses were performed exclusively on pre-aggregated annual national statistics; no individual-level data were accessed.

## Supplementary Materials

The following supporting information can be downloaded at: https://www.mdpi.com/article/10.3390/pathogens15080780/s1, Table S1. Residual and specification diagnostics for the OLS trend and segment models, 2001–2019. DW = Durbin–Watson statistic (values near 2 indicate no first-order autocorrelation); Ljung–Box *p* (lag 4) tests residual autocorrelation; Shapiro–Wilk *p* tests residual normality.
