# Supplementary material for: Epidemiological Trends, Statistical Correlations, and Forecasting of Notifiable Infectious Diseases in South Korea, 2001–2024: A Regression and Time Series Analysis with Projections to 2028
Source: Pathogens. 2026 Jul 23;15(8):780. doi: 10.3390/pathogens15080780 (PMC13516170; doi:10.3390/pathogens15080780)
Supplement: Supplementary file 1 [file pathogens-15-00780-s001.zip › pathogens-4418784-supplementary.pdf]

Supplementary Material

Table S1. Residual and specification diagnostics for the OLS trend and segment models, 2001–2019. DW = Durbin–Watson statistic (values near 2 indicate no first-order autocorrelation); Ljung–Box p (lag 4) tests residual autocorrelation; Shapiro–Wilk p tests residual normality.

Model	DW	Ljung–Box p	Shapiro–Wilk p
Total (excl. 2009)	1.29	0.789	0.011
Total (incl. 2009)	2.05	0.944	<0.001
Incidence rate (excl. 2009)	1.38	0.803	0.008
Class 1	1.38	0.973	<0.001
Class 2	0.96	0.694	0.013
Class 3	1.05	0.215	0.021
Class 4 (excl. 2009–2010)	0.75	0.046	0.182
Class 2 early (2001–2004)	2.25	0.293	0.665
Class 2 growth (2005–2019)	1.75	0.705	0.981

Where the Shapiro–Wilk test flags mild non-normality (driven by discrete count data), negative-binomial log-linear models (Table 2, APC) provide the primary count-appropriate inference; OLS slopes are retained for interpretability.
